# Moxibustion for treating cancer‐related fatigue: A multicenter, assessor‐blinded, randomized controlled clinical trial

**DOI:** 10.1002/cam4.4020

**Published:** 2021-06-29

**Authors:** Kyungsun Han, Mikyung Kim, Eun‐Jung Kim, Yeon‐Cheol Park, Ojin Kwon, Ae‐Ran Kim, Hyo‐Ju Park, Yang‐Chun Park, Jung Hyo Cho, Joo‐Hee Kim, Jun‐Hwan Lee

**Affiliations:** ^1^ Clinical Medicine Division Korea Institute of Oriental Medicine Daejeon Republic of Korea; ^2^ Department of Internal Medicine, College of Korean Medicine Sangji University Wonju Republic of Korea; ^3^ Department of Acupuncture and Moxibustion Medicine, College of Korean Medicine Dongguk University Gyeongju Republic of Korea; ^4^ Department of Acupuncture and Moxibustion Medicine Kyung Hee University Hospital at Gangdong Seoul Republic of Korea; ^5^ Department of Internal Medicine, College of Korean Medicine Daejeon University Daejeon Republic of Korea; ^6^ Department of Acupuncture and Moxibustion Medicine, College of Korean Medicine Sangji University Gangwon‐do Republic of Korea; ^7^ Research Institute of Korean Medicine Sangji University Gangwon‐do Republic of Korea; ^8^ Korean Medicine Life Science University of Science and Technology (UST), Campus of Korea Institute of Oriental Medicine Daejeon Republic of Korea

**Keywords:** cancer‐related fatigue, clinical management, integrative medicine, moxibustion, quality of life, randomized controlled trial

## Abstract

Cancer‐related fatigue (CRF) is one of the most common chronic symptoms experienced by cancer patients. As moxibustion is a popular traditional therapy for managing fatigue, it can be an alternative strategy to treat CRF as well. Therefore, we rigorously designed a full‐scale, multicenter, assessor‐blinded, randomized controlled trial to evaluate the efficacy and safety of moxibustion treatment for CRF. Ninety‐six subjects suffering from CRF were recruited and randomly assigned to moxibustion group, sham moxibustion group, or usual care group. Both the moxibustion group and the sham group received moxibustion treatment for 8 weeks and the usual care group did not. Brief fatigue inventory (BFI) score and Functional Assessment of Cancer Therapy‐Fatigue score were used to assess CRF at baseline and weeks 5, 9, and 13. Questionnaires for the assessment of cognitive impairment, quality of life, and Cold–Heat and Deficiency–Excess patterns were also evaluated. BFI scores significantly decreased in moxibustion group compared to the usual care group (mean difference of −1.92, *p* < 0.001 at week 9 and mean difference of −2.36, *p* < 0.001 at week 13). Although the sham group also showed significant improvement during the treatment period, only the moxibustion group showed improvement after 4 weeks of follow‐up period (mean difference of −1.06, *p* < 0.001). There were no serious adverse events. Our findings confirmed the efficacy and safety of moxibustion for CRF compared to usual care. We also found that moxibustion has a prolonged treatment effect during 4 weeks of follow‐up period.

## Graphical abstract

Moxibustion is widely used treatment method for stimulating acupuncture points and has been used for symptom management in cancer patients in East Asia. However, scientific evidence of moxibustion for the treatment of cancer‐related fatigue is still lacking. Through this full‐scale, multicenter, assessor‐blinded, randomized controlled trial, we found that 8 weeks of moxibustion treatment decreased cancer‐related fatigue more effectively than usual care.

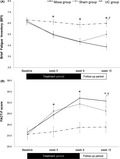

## 1 INTRODUCTION

As advances in early diagnosis and treatment increase the survival rate of cancer patients, the management of cancer survivors is considered as an important issue in addition to cancer treatment. Cancer‐related fatigue (CRF) is one of the most prevalent symptoms in cancer survivors, and it persists chronically after the cancer treatment, which significantly reduces the quality of life.^1^ The prevalence of CRF in cancer patients varies in the literature, but it generally ranges between 40% and 100%.^2^ Despite the high prevalence of CRF in cancer patients, it is often overlooked because it is a common symptom that anyone can experience. What distinguishes CRF from general fatigue is that it cannot be easily overcome even after getting enough rest and that it is irrelevant to the amount of physical activity or severity of the symptoms. CRF is a multifactorial disease caused by a number of mechanisms, including alterations in inflammatory response, neuroendocrine systems, cortisol rhythm, and immune response among others.^1^, ^3^, ^4^ Due to the complexity of underlying mechanisms, there are limited pharmacological interventions to ameliorate CRF.^5^ Therefore, there is an increasing evidence for the use of complementary and integrative interventions to manage CRF, including moxibustion, acupuncture, yoga, and mindfulness, among other techniques.^5^, ^6^, ^7^

In an integrative treatment approach, stimulating sensitized acupoints using acupuncture or moxibustion has been suggested as a potential method to treat physical dysfunction in pain or various diseases.^8^ Studies have suggested that the therapeutic effect of stimulating acupoints is related to nerve segments which may influence the neural–endocrine–immune network and that the therapeutic effect can be increased by selecting appropriate combination of acupoints according to the symptom or disease.^9^, ^10^, ^11^ Acupuncture, the most commonly known method for stimulating acupoints, has been used to treat CRF, and a meta‐analysis showed significant improvement in CRF using acupuncture plus education compared to usual care.^6^ Moxibustion is another widely used method for stimulating acupoints and has been used for symptom management or palliative care in cancer patients.^12^, ^13^ In South Korea, 31.0% of cancer patients are treated with traditional medicine, and moxibustion is one of the most used treatment methods following herbal medicine and acupuncture.^14^

Moxibustion shares its theoretical background with acupuncture but has its own advantages related to thermal stimulation, aromatic effect, herbal effect, and biophysical effects.^15^, ^16^ Traditionally, moxibustion is considered particularly good at supplementing energy, and it is widely applied to people with weakened resilience. Many studies have shown beneficial effects of moxibustion on chronic fatigue,^17^, ^18^, ^19^ and in line with its benefits for managing chronic fatigue syndrome, moxibustion can be a good strategy for managing CRF as well. Several clinical studies have been performed to evaluate therapeutic effect of moxibustion on CRF, showing constantly positive results regardless of moxibustion type.^20^, ^21^, ^22^ There was a systematic review and a meta‐analysis regarding the use of moxibustion for CRF treatment, but the authors found it difficult to draw a conclusion because four RCTs included in the review had high risk of bias and low reporting quality.^23^ Notably, all of the studies included in this systematic review have reported significant improvement in CRF with moxibustion treatment. However, it is important to notice that all four studies performed daily treatment unlike general therapeutic regimen, and none of these studies used sham as controls.^23^ For these reasons, there is a high chance that the expectations for the treatment were reflected in the results. Therefore, we rigorously designed a full‐scale, multicenter, assessor‐blinded, three‐armed, randomized controlled trial to evaluate the efficacy and safety of moxibustion treatment for CRF.

## 2 METHODS

### 2.1 Study design

This was a multicenter, assessor‐blinded, three‐armed, randomized controlled trial. Participants were recruited from three Korean medicine hospitals in South Korea. This study received approval from the institutional review board of each hospital. The trial was registered at Clinical Research Information Service (CRIS) before the study (CRIS identifier: KCT0002170). The study was conducted from 27 February 2017 to 5 February 2018. Those who voluntarily signed the informed consent after receiving an explanation about the trial underwent eligibility screening. The study participants were randomly allocated to one of the three groups. Outcome measures were assessed before the initial intervention and at weeks 5 and 9 after completing 8 weeks of intervention. The follow‐up assessment was done at week 13. Complete details of the trial protocol have been published previously.^24^

### 2.2 Sample size calculation

As moxibustion and acupuncture share common treatment methods that stimulate acupuncture points, the adequate sample size was estimated based on a prior acupuncture study on the same disease that used BFI score as a primary outcome.^25^ The estimated mean difference and common standard deviation between moxibustion and sham moxibustion group (mean difference = −1.6, SD = 2.0) were used to determine the sample size. For three arms (moxibustion group, sham moxibustion group, and usual care group), 32 participants were required per group to achieve 80% power at 5% level of significance (two‐sided) and an anticipated dropout rate of 20%.

### 2.3 Participants

A total of 96 participants suffering from CRF were recruited. Participants were recruited through online and offline notice boards of the hospitals, local newspapers, flyers, and advertisement boards at public places. On their first visit, written informed consent was obtained from each participant after thoroughly explaining the study process. Among volunteers who had spontaneously provided the written informed consent, eligibility was determined based on the inclusion and exclusion criteria. Male and female participants aged 19–79 years whose cancer‐related treatment ended at least 12 weeks before the trial (hormone therapy that has been going on for more than three weeks was allowed) were enrolled in this study. Furthermore, we included participants with continuous fatigue related to cancer treatment or cancer itself for at least 4 weeks, those who met the ICD‐10‐CM diagnostic criteria for CRF, those who had BFI score more than 4, and those who had Eastern Cooperative Oncology Group (ECOG) performance status of ≤2. Participants were excluded if they (1) had experienced fatigue before the diagnosis of cancer, (2) had severe anemia (platelet count <60,000/μl, hemoglobin <8g/dl, or absolute neutrophil count <1000/μl), (3) had ongoing aggressive treatment for anemia (e.g., erythropoietin or blood transfusion), (4) had poor oral intake with a less‐than‐normal level of serum albumin, (5) had any significant sign or symptom of inflammation with C‐reactive protein (CRP) ≥10 mg/L and white blood cell >10,000/μl, (6) had abnormal findings in a thyroid function test (abnormal level of free thyroxine and thyroid stimulating hormone <0.1 μIU/ml or TSH >5.1 μIU/ml), (7) had abnormal findings in a liver function test or renal function test (aspartate aminotransferase or alanine aminotransferase more than two times the upper normal range or creatinine ≥2.0 mg/dl) or the presence of serious liver or renal failure, (8) had a core of 11 points or more on either the anxiety or depression subscale of the Hospital Anxiety and Depression Scale (HADS), (9) had an insomnia severity index (ISI) score of 15 points or more, (10) had the level of cancer‐related pain measured by the numeric rating scale ≥4, (11) had an estimated life expectancy of six months or less, (12) had a plan to undergo surgery, chemotherapy, or radiotherapy during the study, (13) had a history of medication (methylphenidate, modafinil, burpropion, or dexamethasone) to manage CRF taken within 4 weeks, (14) had a history of Korean medical treatment to manage CRF within 4 weeks, (15) had initiated or had changes in dietary supplement regimen or non‐pharmacologic therapies for alleviating CRF during the trial or within 4 weeks, (16) had participated in other clinical trials within 4 weeks, (17) had a history of hypersensitivity reactions or serious adverse reactions after moxibustion treatment or had problems in cooperating with thee moxibustion treatment due to other reasons, such as dyspnea, and (18) had other apparent factors or any diseases that could cause current fatigue other than cancer treatment or the cancer itself. This study also excluded women who were pregnant, lactating or planning to become pregnant during the study period.

### 2.4 Interventions

There were three groups in this study: the moxibustion (Mx) group, sham moxibustion (SMx) group, and usual care (UC) group. The initiation of any new treatment used to attenuate CRF was prohibited during the study, except for non‐pharmacologic therapies, such as exercise, yoga, and meditation or dietary supplements that were in continuation since more than 4 weeks before the trial.

Moxibustion and sham moxibustion treatments were conducted by certified Korean medicine doctors using two types of moxibustion devices: the ignition‐type moxibustion device (IM) and the electrical moxibustion apparatus (EM). For the Mx group or SMx group, subjects received 30 min treatments twice per week for 8 weeks. Participants in the Mx group received treatment with two IMs on the abdomen area, CV8 (*Shenque*) and CV12 (*Zhongwan*), and four EMs attached at LI4 (*Hegu*) and ST36 (*Zusanli*), bilaterally. The SMx group received treatment at non‐acupoints, which were points irrelevant to addressing fatigue, with sham devices. For the SMx group, two sham IMs were applied on the abdomen (approximately 3 cm on both sides from 13.5 cm above the umbilicus) and four sham EMs were attached on upper limbs (1 cm lateral and 5 cm distal to the cubital creases of the forearms) and lower limbs (upper 1/3 points of the medial line of the tibia). The SMx devices were designed to exclude the therapeutic effect while maintaining blinding as much as possible. We confirmed that the skin temperature rose to 45°C during the EM treatment, thus making the participants feel slightly warm on their skin; however, the temperature did not sufficiently rise to stimulate the acupoints. Detailed protocol for moxibustion has been published previously.^24^ The participants in the UC group did not receive moxibustion treatment and maintained their usual treatment and self‐care. Regardless of their allocated groups, all participants were provided a brochure on the management of CRF.

### 2.5 Randomization and blinding

Randomization was done by an independent statistician using SAS® Version 9.4. Random numbers were sealed in opaque envelopes and kept in a double‐locked cabinet. The practitioners of each research center opened envelops to see which group their subjects were allocated to. Outcome assessment was done by independent researchers who were not involved in the treatment procedure. Therefore, although practitioners cannot be blinded, assessors were blinded to all three groups.

The appearance of the moxibustion devices used for the SMx group were exactly the same as those used for the Mx group. This was to maintain the blinding among participants in Mx and SMx groups. The participants could see the practitioners ignite the mugwort for IM. During the treatment, participants could see small amounts of smoke and smell the emanating odor. However, the base of the sham IM was filled with an insulator to block heat. Regarding EMs, the participants could see the EM attached on their skin with a blinking light on the top of the device so that it could not be distinguished by eyes. To assess whether the blinding process was successfully achieved in Mx and SMx groups, blinding and credibility tests were assessed after the initial and final treatments.

### 2.6 Outcome measures

Independent researchers who were blinded to the group allocation and were not involved in the intervention assessed the outcome measures. The primary endpoint of this study was week 9, which was after the 8‐week intervention, and the primary outcome of this study was the mean change in the Brief Fatigue Inventory (BFI) score.^26^, ^27^ BFI is a simple measurement of fatigue which comprises nine questions about the degree of fatigue. A higher BFI score indicates severe fatigue symptoms: 1–3 points indicate mild fatigue, 4–6 points indicate moderate fatigue, and 7–10 points indicate severe fatigue.^28^ The Functional Assessment of Cancer Therapy‐Fatigue (FACT‐F) questionnaire was used to assess CRF as a secondary outcome. FACT‐F is a multidimensional fatigue assessment tool with 13 items to assess multiple fatigue characteristics and its related function.^29^ Score ranges from 0 to 52 with lower score indicating greater fatigue symptoms. BFI and FACT‐F were assessed at the baseline and at weeks 5, 9, and 13.

We used the Korean version of the European Organization for Research and Treatment of Cancer (EORTC) core quality of life questionnaire version 3.0 (QLQ‐C30) to assess the quality of life of cancer patients.^30^ This questionnaire comprises 30 questions specifically related to cancer and its treatment, and it is subdivided into three categories: global health status, functional scales, and symptom scales.^31^, ^32^ High global health status and functional scale scores represent a higher quality of life, whereas a high score for symptom scales represents a lower quality of life. This questionnaire was administered at weeks 0, 9, and 13. To assess whether fatigue affected cognitive function, we used Korean version of Montreal Cognitive Assessment (MoCA‐K) for assessing the level of cognition at weeks 0 and 9.^33^ It is a sensitive screening instrument to detect mild cognitive impairment in various diseases as well as Alzheimer's disease. Although performance on the MoCA varies among populations, such as age, ethnicity, and education, a score of 26 or above is generally considered normal and the MoCA‐K cutoff score of ≤22 is used to screen for mild cognitive impairment.^34^, ^35^

For safety assessment, blood pressure and body temperature were evaluated at every visit while adverse events were continuously monitored throughout the study. The laboratory test was done at the screening visit and week 9.

### 2.7 Pattern identification based on Korean traditional medicine

For subgroup analyses, a questionnaire for Cold–Heat & Deficiency–Excess pattern identification (CHDE) was done at the baseline. The reason for subgroup analyses was to provide scientific evidence for pattern identification as it is an important factor for selecting the appropriate treatment in traditional Chinese or Korean medicine. CHDE is a validated questionnaire that comprises 34 questions (nine questions for the cold pattern, six questions for the heat pattern, 13 questions for the deficiency pattern, and six questions for the excess pattern) in the format of a typical five‐level Likert scale.^36^ By adding scores of each category, participants were categorized into dominant pattern, either the cold or the heat and either the deficiency or the excess pattern.

### 2.8 Statistical analyses

Statistical analysis was done by an independent statistician blinded to group allocation as described previously.^24^ Full analysis set (FAS) was used for the analysis, and the multiple imputation method was adopted for incomplete data sets. Using SAS® Version 9.4 (SAS institute. Inc), summary statistics of baseline characteristics of each group are presented as the mean and 95% confidence intervals (CI). For qualitative data, Fisher's exact test was performed. To validate changes in the BFI scores between groups, we assumed two sets of null hypotheses: (1) There is no difference in the mean change of the BFI before and after the treatment between the Mx group and the UC group; (2) There is no difference in the mean change of the BFI before and after the treatment between the Mx group and the SMx group. To verify the hypotheses, analysis of covariance was performed to explore between‐group differences for the outcome variables with the baseline values as a covariate and each group as a fixed factor. Paired *t*‐test was used for within‐group comparisons. *p* values less than 0.05 in two‐sided test were considered statistically significant in this study.

## 3 RESULTS

### 3.1 Demographics and characteristics of participants

**FIGURE 1:**
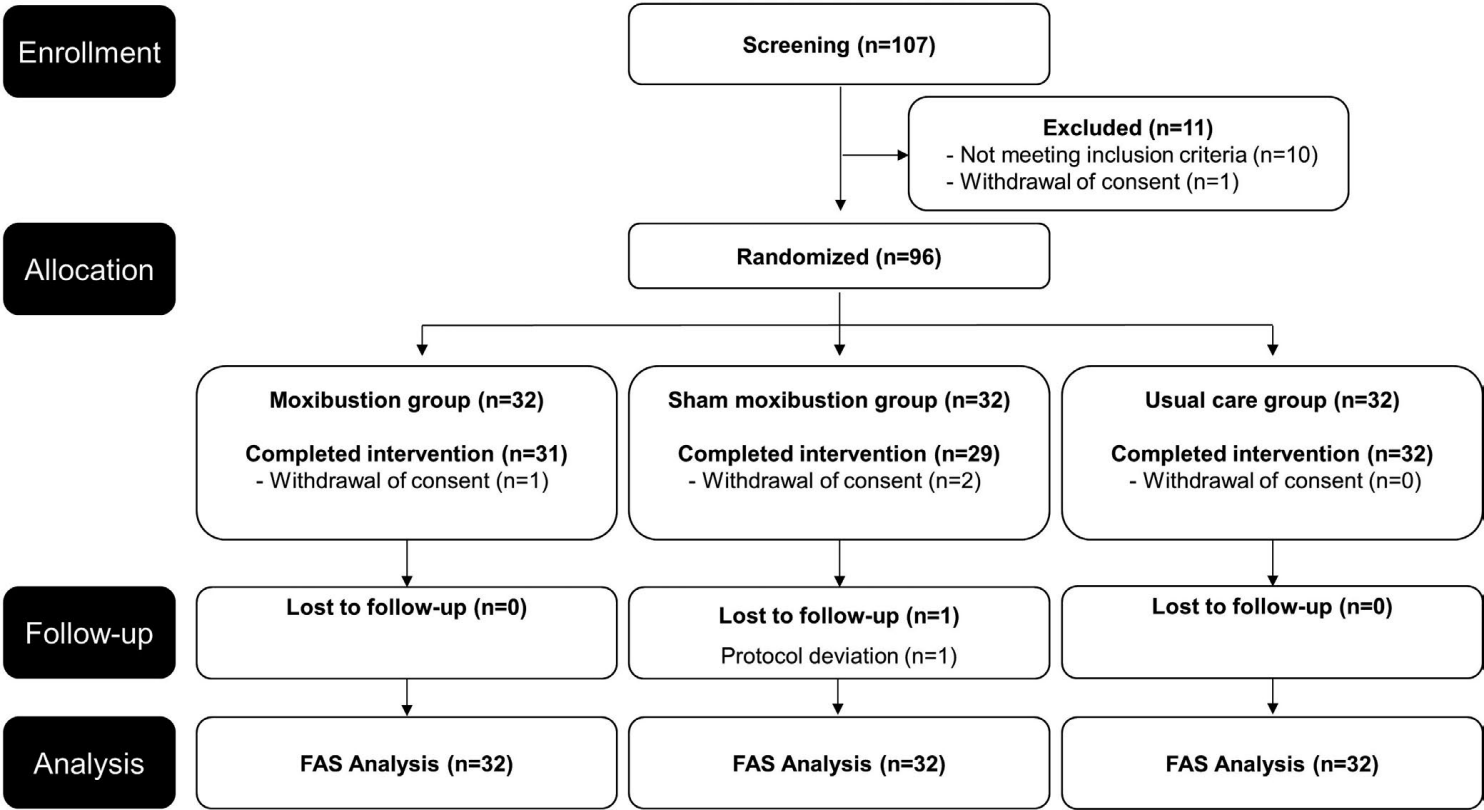
Study flow diagram

We screened 107 volunteers and assessed them for eligibility. Ultimately, 96 participants were enrolled into the study (Figure 1). Participants were randomly allocated to the Mx group, the SMx group, or the UC group. A total of 92 participants completed the study. One participant in the Mx group and two participants in the SMx group withdrew their consent during the intervention for personal reasons. In addition, one participant from the SMx group dropped‐out during the follow‐up period due to deviation from the protocol. After the intervention, the questionnaire was administered to confirm if the blinding was appropriate for the SMx group. In the Mx group, 70.97% of the subjects thought they had been treated with real moxibustion, 6.45% thought they were enrolled in the sham group, and 22.58% of the patients answered that they did not know which group they were enrolled in. In the SMx group, 59.38% of participants thought they had received real treatment, 18.75% thought they had received the sham treatment, and 21.88% of the patients answered that they did not know which group they were enrolled in. Blinding tests showed no statistical differences between the two groups (*p* = 0.3918, Table S1). Among the three groups, there was no difference observed in the baseline characteristics (Table 1). Overall, the majority of the study subjects were women (78.1%), were non‐smokers (99.0%), were nonalcoholic individuals (88.5%), and had an average of 51 months passed since cancer diagnosis.

**TABLE 1:** Baseline characteristics of this study

Characteristics	Mx group (n = 32)	UC group (n = 32)	SMx group (n = 32)	*p*
Gender^†^				
Male	5 (15.6%)	7 (21.9%)	9 (28.1%)	0.532
Female	27 (84.4%)	25 (78.1%)	23 (71.9%)	
Age (years)^‡^	53.03 (49.78, 56.28)	56.06 (52.96, 59.17)	56.44 (52.91, 59.96)	0.268
Height (cm)^‡^	160.16 (157.41, 162.90)	162.75 (160.09, 165.42)	161.57 (159.30, 163.85)	0.349
Weight (kg)^‡^	56.88 (53.87, 59.88)	59.39 (56.15, 62.62)	59.65 (56.54, 62.76)	0.372
Smoking^†^				
Yes	0 (0.0%)	0 (0.0%)	1 (3.1%)	
No	32 (100.0%)	32 (100.0%)	31 (96.9%)	1.000
Alcohol^†^				
Yes	5 (15.6%)	5 (15.6%)	1 (3.1%)	
No	27 (84.4%)	27 (84.4%)	31 (96.9%)	0.217
Exercise^†^				
Yes	26 (81.3%)	28 (87.5%)	28 (87.5%)	
No	6 (18.7%)	4 (12.5%)	4 (12.5%)	0.817
Currently employed^†^				
Yes	10 (31.3%)	11 (34.4%)	8 (25.0%)	
No	22 (68.8%)	21 (69.8%)	24 (75.0%)	0.708
Cancer type^†^				
Breast cancer	12 (37.5%)	12 (37.5%)	13 (40.6%)	
Other cancers	20 (62.5%)	20 (62.5%)	19 (59.4%)	0.957
Cancer stage^†^				
0	3 (9.4%)	2 (6.3%)	1 (3.1%)	
I	7 (21.9%)	7 (21.9%)	6 (18.8%)	
II	7 (21.9%)	12 (37.5%)	11 (34.4%)	
III	9 (28.1%)	4 (12.5%)	7 (21.9%)	
IV	0 (0.0%)	0 (0.0%)	2 (6.3%)	
Unknown	6 (18.8%)	7 (21.9%)	5 (15.6%)	0.558
Previous treatments^†^				
Surgery	32 (100.0%)	31 (96.9%)	30 (93.8%)	0.356
Chemotherapy	18 (56.3%)	19 (59.4%)	21(65.6%)	0.737
Radiation therapy	10 (31.3%)	13 (40.6%)	17 (53.1%)	0.205
Time from diagnosis (months)^‡^	48.16 (29.84, 66.48)	49.94 (32.66, 67.21)	54.28 (40.53, 68.04)	0.860
CHED patterns^†^				
Cold	28 (87.5%)	28 (87.5%)	26 (81.25%)	0.817
Heat	4 (12.5%)	4 (12.5%)	6 (18.75%)	
Excess	15 (46.9%)	13 (40.6%)	18 (56.25%)	0.491
Deficiency	17 (53.1%)	19 (59.4%)	14 (43.75%)	

Data shown as mean (95% Confidence Interval) or frequency (percentage).

† Fisher's exact test.

‡ Analysis of variance.

### 3.2 Cancer‐related fatigue

**FIGURE 2:**
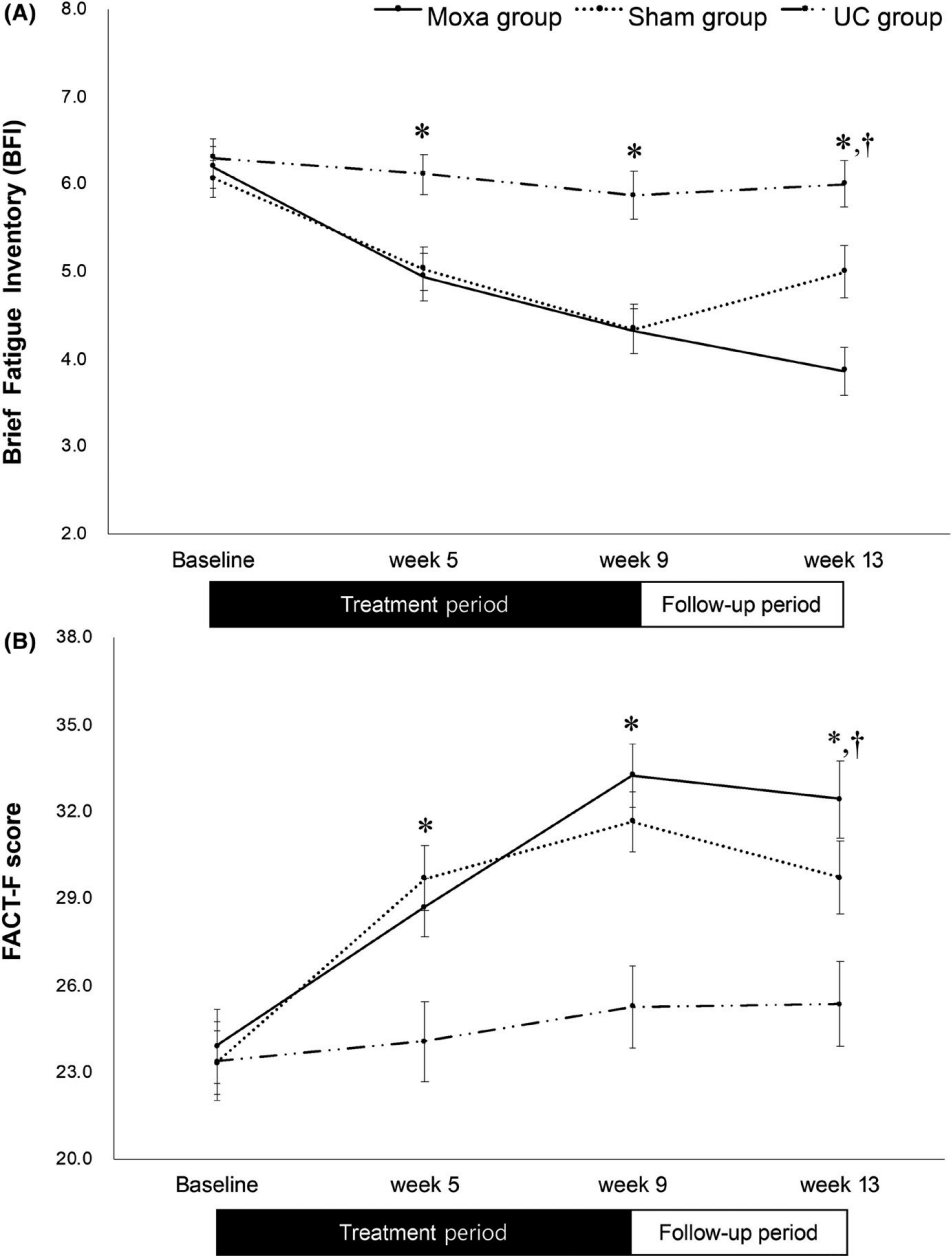
Changes of the fatigue symptoms. (A) Changes of the Brief Fatigue Inventory (BFI) scores. (B) Changes of Functional Assessment of Cancer Therapy‐Fatigue (FACT‐F) scores. Mx group: moxibustion group; SMx group: sham moxibustion group; UC group: usual care group; *: statistically significant (*p* < 0.05) between the Mx group and the UC group. †: statistically significant (*p* < 0.05) between the Mx and the SMx group

The primary outcome of this study— the BFI score —measures the severity of fatigue during the past 24 hours. The BFI score in the Mx group significantly decreased from the baseline to week 9 (mean difference of −1.92, *p* < 0.001) and week 13 (mean difference of −2.36, *p* < 0.001). Similar changes were noted for the SMx group (Table 2, Figure 2A). The BFI score decreased from the baseline to week 9 (mean difference of −1.71, *p* < 0.001) and week 13 (mean difference of −1.08, *p* = 0.001). Although all three groups experienced a significant improvement in the BFI score at week 9, the Mx group showed the greatest improvement which lasted for the post‐intervention period. In between‐group comparisons, there were significant differences between the Mx group and the UC group at week 5 (mean difference of −0.58, *p* < 0.001), week 9 (mean difference of = −0.76, *p* < 0.001), and week 13 (mean difference of −1.06, *p* < 0.001). The differences between the Mx group and the SMx group were significant only at week 13 (mean difference of −1.17, *p* = 0.004). Similar trends were observed with the FACT‐F scores, another self‐reported scale for the CRF. Both the Mx group and the SMx group showed significant improvement during the intervention, and the UC group did not show any statistically significant improvement in the FACT‐F score (Table 2, Figure 2B). The MoCA‐K score in all three groups significantly increased after the intervention (Table 2).

**TABLE 2:** Questionnaires for the assessment of fatigue symptoms and cognitive impairment

	Mx group (n = 32)	UC group (n = 32)			SMx group (n = 32)		
	Mean (95% CI)	Mean (95% CI)	Mean difference Mx‐UC (95% CI)	*p* ^†^	Mean (95% CI)	Mean difference Mx‐SMx (95% CI)	*p* ^†^
BFI							
Baseline	6.19 (5.71, 6.67)	6.30 (5.86, 6.74)			6.06 (5.64, 6.49)		
Week 9	4.27 (3.77, 4.76)	5.87 (5.32, 6.41)	−0.76 (−1.06, −0.47)	<0.001*	4.34 (3.80, 4.90)	−0.13 (−0.83, 0.57)	0.712
*p* ^‡^	<0.001*	0.036*			<0.001*		
Week 13	3.83 (3.28, 4.37)	6.00 (5.46, 6.53)	−1.06 (−1.41, −0.71)	<0.001*	4.98 (4.40, 5.55)	−1.17 (−1.96, −0.39)	0.004*
*p* ^‡^	<0.001*	0.186			0.001*		
FACT‐F							
Baseline	23.91 (21.30, 26.51)	23.88 (21.09, 26.66)			23.31 (21.09, 25.54)		
Week 9	33.25 (31.09, 35.41)	25.25 (22.33, 28.17)	3.99 (2.49, 5.50)	<0.001*	31.65 (29.52, 33.78)	1.47 (−1.51, 4.45)	0.334
*p* ^‡^	<0.001*	0.152			<0.001*		
Week 13	32.42 (29.71, 35.13)	25.34 (22.32, 28.37)	3.53 (1.89, 5.16)	<0.001*	29.72 (27.15, 32.29)	2.43 (−0.97, 5.84)	0.161
*p* ^‡^	<0.001*	0.135			<0.001*		
MoCA‐K							
Baseline	27.41 (26.65, 28.16)	26.97 (26.23, 27.71)			25.53 (24.39, 26.67)		
Week 9	28.41 (27.88, 28.95)	28.16 (27.60, 28.71)	0.04 (−0.28, 0.36)	0.820	27.13 (26.12, 28.15)	0.07 (−0.75, 0.89)	0.861
*p* ^‡^	0.001*	0.001*			<0.001*		

Data shown as mean (95% Confidence Interval). BFI, brief fatigue inventory; FACT‐F, Functional Assessment of Cancer Therapy‐Fatigue; MoCA‐K, Korean version of Montreal cognitive assessment.

* *p* value less than 0.05 was considered statistically significant.

† *p* value obtained from group comparisons.

‡ *p* value obtained from within‐group comparisons (compared to baseline value).

### 3.3 Quality of Life

Significant improvement in global health status was observed in both Mx and SMx groups compared to the UC group, but there were no statistically significant differences between Mx group and the SMx group (Table 3). Among the functional scales of QLQ C‐30, all five scales significantly improved in the Mx group, but emotional and cognitive function scores did not statistically significantly increase in the SMx group (Table S2). In group comparison with the UC group, the Mx group showed significant improvement in physical, emotional, and cognitive functions. In symptom categories, the overall score decreased in all three groups, with the Mx group showing the greatest improvement. Compared to the UC group, the Mx group showed significant improvement in fatigue, dyspnea, appetite loss, and diarrhea after moxibustion. Compared to the SMx group, only the symptom score of diarrhea significantly improved in the Mx group (*p* value of 0.019 in week 9 and 0.035 in week 13).

**TABLE 3:** Mean difference in quality of life assessments with EORTC QLQ‐C30 scores before and after the 8‐week intervention

Variables	Mean difference Mx‐UC (95% CI)	*p*	Mean difference Mx‐SMx (95% CI)	*p*
Global health status				
Quality of Life	5.30 (1.55, 9.05)	0.006*	−0.80 (−8.82, 7.22)	0.847
Functional scales				
Physical	5.15 (2.76, 7.53)	<0.001*	4.36 (−1.22, 9.93)	0.125
Role	4.29 (−0.21, 8.78)	0.062	1.10 (−6.80, 8.99)	0.786
Emotion	4.60 (0.45, 8.76)	0.030*	5.39 (−3.49, 14.26)	0.234
Cognitive	5.65 (1.38, 9.93)	0.010*	2.83 (−6.27, 11.93)	0.543
Social	3.29 (−1.52, 8.09)	0.180	0.15 (−10.22, 10.52)	0.978
Symptom scales				
Fatigue	−7.57 (−11.54, −3.60)	0.001*	−1.50 (−9.04, 6.03)	0.695
Nausea/Vomiting	−0.54 (−3.87, 2.80)	0.753	2.84 (−3.32, 9.01)	0.366
Pain	−2.67 (−6.96, 1.62)	0.222	−2.28 (−10.64, 6.08)	0.592
Dyspnea	−5.16 (−9.72, −0.61)	0.026*	−5.65 (−15.43, 4.14)	0.258
Sleeping disturbances	−4.55 (−10.38, 1.28)	0.126	−2.31 (−11.66, 7.04)	0.629
Appetite Loss	−6.59 (−11.22, −1.96)	0.005*	−9.72 (−20.52, 1.08)	0.078
Constipation	−0.81 (−4.74, 3.11)	0.685	5.40 (−3.25, 14.05)	0.221
Diarrhea	−5.02 (−9.27, −0.78)	0.020*	−8.62 (−15.82, −1.43)	0.019*
Financial difficulties	−1.19 (−5.71, 3.33)	0.606	2.03 (−7.19, 11.26)	0.666

Mx, moxibustion group; UC, usual care group; SMx, sham moxibustion group

* *p* value less than 0.05 was considered statistically significant.

### 3.4 Safety assessments

For safety assessments, blood pressure and body temperature were evaluated at every visit. Blood tests were done at the baseline and week 9 to confirm participants’ safety. There were no significant differences in vital signs and blood analysis (Tables S3 and S4). No serious adverse events occurred throughout the study. Nine participants in the Mx group, 11 participants in the SMx group, and one participant in the UC group reported minor adverse events (*p* = 0.0192 from Chi‐square test). The most common adverse events were dyspepsia and upper respiratory infection. Although most adverse events were unrelated to the intervention, two participants in the Mx group reported mild burns at the moxibustion site. Mild burns were managed with adequate dressing and a burn ointment.

### 3.5 CHDE pattern identification

In all three groups, there were more patients with cold pattern than heat pattern, but the distribution of excess and deficiency patterns was similar (Table 1). To understand the impact of the CHDE pattern identification in relation to CRF, difference of treatment effect was compared according to whether subjects had CHDE symptoms (Table 4). Compared to the UC group, moxibustion was more effective in the CRF patients with cold pattern, deficiency pattern, and excess pattern at weeks 9 and 13. Compared to the SMx group, moxibustion showed statistically greater improvement in BFI score in patients with cold pattern and excess pattern only at week 13.

**TABLE 4:** Subgroup analysis according to the Cold–Heat & Deficiency‐Excess pattern identification according to the traditional medicine theory

	Brief Fatigue Inventory (BFI) score			
	Mean difference (Mx–UC)	*p*	Mean difference (Mx–SMx)	*p*
Cold pattern				
Week 9	−1.64 (−2.27, −1.00)	<0.001*	−0.06 (−0.83, 0.70)	0.870
Week 13	−2.16 (−2.96, −1.36)	<0.001*	−1.21 (−2.10, −0.32)	0.009*
Heat pattern				
Week 9	−0.56 (−2.71, 1.60)	0.561	0.35 (−2.33, 3.04)	0.751
Week 13	−1.46 (−3.41, 0.50)	0.121	−0.40 (−3.76, 2.96)	0.772
Deficiency pattern				
Week 9	−1.50 (−2.49, 0.52)	0.004*	−0.51 (−1.62, 0.60)	0.355
Week 13	−1.93 (−3.14, −0.73)	0.003*	−1.04 (−2.25, 0.18)	0.092
Excess pattern				
Week 9	−1.54 (−2.33, −0.76)	<0.001*	0.41 (−0.56, 1.37)	0.394
Week 13	−2.35 (−3.27, −1.43)	<0.001*	−1.31 (−2.52, −0.11)	0.034*

Data shown as mean differences (95% Confidence Interval). Mx, moxibustion group; UC, usual care group; SMx, sham moxibustion group.

* *p* value less than 0.05 was considered statistically significant.

## 4 DISCUSSION

To our knowledge, this is the first rigorously designed full‐scale clinical trial to evaluate the efficacy and safety of moxibustion for CRF. We have successfully blinded real and sham moxibustion by appearance and differentiated the stimulation by selecting acupoints versus non‐acupoints and by varying the temperature stimulus. In this study, we compared moxibustion with sham moxibustion and usual care for 8 weeks plus 4 weeks of follow‐up period. The results showed that the Mx group showed significantly alleviated fatigue and improved quality of life compared to the UC group. However, the SMx group also showed significant improvement in the BFI score compared to the UC group, and therefore, there was no significant difference between the Mx group and the SMx group at the end of the treatment session. As moxibustion has traditionally been applied to various chronic diseases, including chronic fatigue, there have been numerous studies on its treatment mechanism. These studies have suggested that it may involve modulation of the autonomic nervous system, hypothalamic–pituitary–adrenal axis, and antioxidative activity.^17^, ^37^, ^38^, ^39^ In addition, there have been attempts to elucidate the treatment mechanism of moxibustion for fatigue in animal studies, which have suggested that it may be related to modulation of inflammatory cytokines and neurotransmitters, such as 5‐hydroxyteyptamine and dopamine, in the hippocampus and reduced serum malonaldehyde levels.^9^, ^40^

When evaluating the therapeutic effect in CRF patients, placebo response is an important factor to consider.^41^ The placebo response rate of CRF patients reportedly varies from 27% to 60%, and if the subjects are women with higher anxiety at baseline, placebo response will be higher.^42^ It is known that even open‐label placebo improves symptoms in CRF patients.^43^ Therefore, in preliminary studies of moxibustion for CRF, most studies used usual care as the control.^23^ Taking the placebo effect into account, we designed a three‐arm study. As expected, both the Mx group and the SMx group alleviated CRF symptoms during the treatment period, even though the degree of improvement was greater in the Mx group. However, the most interesting finding in this study is that although both these groups showed significant improvement, only the Mx group showed improvement after 4 weeks of follow‐up. There is no clear evidence for the prolonged effect of moxibustion, but there was a clinical trial suggesting that the mechanism underlying long‐term moxibustion treatment for fatigue alleviation may be related to modulation of the vagus nerve.^39^ The study compared the different effects of acupuncture and moxibustion for chronic fatigue syndrome; the comparison revealed that although acupuncture was better for instantaneous changes in heart rate variability (HRV), moxibustion was more effective in the long‐term modulation of HRV.^39^

Improvement in the overall quality of life is also an important factor in treating CRF patients because unusual tiredness can severely affect patients’ quality of life and survival rate and can also be a strong predictor of cancer survival.^44^ In the current study, EORTC QLQ‐C30 showed a similar tendency with fatigue scales. Significant improvement in global health status was observed in both the Mx group and the SMx group compared to the UC group. The Mx group showed significant improvement in fatigue, dyspnea, appetite loss, and diarrhea scores compared to the UC group. Compared to the SMx group, diarrhea symptoms improved in the Mx group. There are growing evidence showing that moxibustion can alleviate diarrhea.^45^, ^46^ An RCT study of moxibustion for treating anorexia with metastatic cancer which shares almost the same acupoints as our study (CV8, CV12, CV4, and ST36) showed significant alleviation of anorexia and improvement in the quality of life compared to placebo moxibustion.^47^ Putting together, moxibustion may be used to improve the QOL in the management of cancer patients, particularly in patients with gastrointestinal symptoms, such as anorexia and diarrhea.

Patients with chronic fatigue often complain of cognitive impairment described as “brain fog,” and numerous studies have shown that chronic fatigue can adversely affect cognitive functions of cancer patients.^48^, ^49^ Conversely, some patients receiving brain radiation treatment can experience cognitive decline that could cause fatigue.^50^ To distinguish CRF from mild cognitive impairment, we included MoCA‐K as a secondary outcome measure. It is a promising tool for screening cancer‐related cognitive impairment.^51^ In our study results, the baseline values for the cognitive function, as indicated by the MoCA‐K score, were all within the normal range, which is above 22. Predictably, there were no statistically significant differences in the MoCA‐K score after the treatment.

In traditional Chinese medicine or traditional Korean medicine theory, moxibustion is primarily used in deficiency/cold syndrome due to its warming and nourishing effects; however, it is also applied in excess/heat syndrome.^15^ In terms of the cold–heat pattern, our results showed that moxibustion was more effective in the cold pattern than the heat pattern. The results were predictable because patients with the heat pattern tend to avoid heat stimulation, even though there is no difference in the actual body temperature between the cold–heat patterns.^52^ Interestingly, our study showed that moxibustion can be applied for both deficiency and excess patterns. Moxibustion is generally considered a treatment suitable for the deficiency pattern because it has been used in chronic diseases and considered an energy‐supplementing treatment in traditional medicine. In a cross‐sectional study in Taiwan, CRF was related to Yang‐deficiency and Qi‐deficiency in traditional Chinese medicine body constitution, and the authors suggested that pattern identification can be useful for selecting appropriate treatment for CRF patients.^53^ However, moxibustion can have therapeutic effects on both the excess pattern and the deficiency pattern by promoting the circulation in the Meridian system based on the traditional medicine theory, which in turn regulates the balance between Yin and Yang.^16^ In the same context, there was a study showing that local thermotherapy can reduce the stress response by increasing parasympathetic nervous activity while decreasing sympathetic nervous activity.^54^ Putting together, moxibustion may have had therapeutic effects in both the excess and the deficiency patterns by regulating the balance of autonomic nervous activity; however, the exact mechanism is unknown. Further studies to explore therapeutic mechanisms of moxibustion are needed.

There are some limitations in this study. Although this was a multi‐center trial, the study was conducted in three sites only in South Korea, which may limit the generalizability of this study's results. Although our findings showed that it is generally safe to apply moxibustion to CRF patients, it is important to note that the current study was particularly focused on chronic CRF, and patients who had a plan of surgery, chemotherapy, or radiotherapy during the study were excluded. Therefore, applying moxibustion in practice needs caution depending on the stage and location of the cancer. Further study on the safety and effectiveness of moxibustion for acute fatigue symptoms during or right after cancer treatment is needed. Another limitation was that the follow‐up period of our study was as short as 4 weeks, and thus, longer‐term studies are needed to explore the persistent effects of the moxibustion. As we already mentioned above, our results failed to prove that moxibustion is superior to placebo although there was an interesting prolonged effect of moxibustion during the follow‐up period. To further maximize the effect of moxibustion treatment while reflecting the practical treatment methods, more elaborate RCT design is needed in future studies. Taking each subject's symptoms into account, allowing the use of individualized acupoints along with fixed acupoints, or including symptom patterns suitable for moxibustion treatment in inclusion criteria may be helpful.

In conclusion, our study confirmed the efficacy and safety of moxibustion for CRF compared to usual care. Compared to the sham group, although there was no significant difference during the treatment period, moxibustion showed a significantly prolonged effect after 4 weeks of the follow‐up period. In a subgroup analysis using the Korean traditional CHED patterns, we found that moxibustion was effective in all groups except for the heat pattern group. Further study using objective biomarkers of CHED patterns is needed to explore the therapeutic mechanisms of moxibustion in CRF patients. For patients and clinicians, this novel approach offers an alternative management strategy for CRF, which is likely to be effective and safe.

## 5 ETHICAL APPROVAL STATEMENT AND CLINICAL TRIAL REGISTRATION NUMBER

This study was conducted according to the principles of Declaration of Helsinki and good clinical practice guidelines. This study received approval from the institutional review board of each hospital; Daejeon Oriental Hospital of Daejeon university (approval no. DJOMC‐141‐1), Kyung Hee University oriental hospital at Gangdong (approval no. KHNMCOH 2016‐09‐004), and Dongguk university Bundang oriental hospital (approval no. 2016‐0008). The trial was registered at Clinical Research Information Service (CRIS) before the study (CRIS identifier: KCT0002170).

## CONFLICT OF INTEREST

The authors declared no potential conflicts of interest with respect to the research, authorship, and/or publication of this article.

## AUTHOR'S CONTRIBUTIONS

KH is responsible for the data analysis and drafted the manuscript. OK planned the statistical strategy and was actively involved in the sample size calculation and random allocation. JHL is the principal investigator of the research project. KJH and LJH contributed to conception of the study therefore JHK and JHL have the final responsibility for publication. MK, EJK, YCP, YCP, JHC, JHK, and JHL took part in the study design. As investigators at the hospital, KH, MK, EJK, YCP, ARK, HJP, YCP, JHC, and JHK were involved in the protocol development and IRB approval. All authors have read, revised, and approved the final version of the manuscript.

## Supporting information

Table S1‐S4

## Data Availability

The data used to support the findings of this study are available from the corresponding author upon request.
