# Supplementary material for: Moxibustion for treating cancer‐related fatigue: A multicenter, assessor‐blinded, randomized controlled clinical trial
Source: Cancer Med. 2021 Jun 29;10(14):4721–33. doi: 10.1002/cam4.4020 (PMC8290232; doi:10.1002/cam4.4020)
Supplement: Supplementary file 1 — Table S1‐S4 [file CAM4-10-4721-s001.docx]

Supplementary tables

**Supplementary table 1. Blinding test results**

|  | Moxibustion group | Sham moxibustion group | *p* ^†^ |
| --- | --- | --- | --- |
| Moxibustion | 22 (70.97%) | 19 (59.38%) | 0.3918 |
| Sham moxibustion | 2 (6.45%) | 6 (18.75%) |  |
| Don`t know | 7 (22.58%) | 7 (21.88%) |  |
| New Blind Index | 0.645 (0.435, 0.856) | -0.406 (-0.678, -0.134) |  |
| † Chi-square test |  |  |  |

**Supplementary Table 2. Changes in the quality of life assessed with EORTC QLQ-C30 scores**

|  | Variables | | Mx group  (n=32) | UC group  (n=32) | *p* | SMx group  (n=32) | *p* |
| --- | --- | --- | --- | --- | --- | --- | --- |
| **Global health status / QoL** | | | | | | | |
|  | Qol | Baseline | 47.13  (41.54, 52.71) | 46.35  (39.83, 52.87) |  | 43.75  (38.14, 49.36) |  |
|  |  | week 9 | 60.93  (55.36, 66.49) | 50.00  (43.66, 56.34) | **0.006*** | 60.59  (54.39, 66.78) | 0.845 |
| **Functional scales** | | | | | | | |
|  | Physical | Baseline | 64.38  (59.24, 69.51) | 65.41  (59.97, 70.86) |  | 66.24  (61.56, 70.93) |  |
|  |  | week 9 | 78.63  (74.96, 82.30) | 68.75  (64.45, 73.04) | **<.001*** | 75.08  (70.00, 80.16) | 0.125 |
|  | Role | Baseline | 64.07  (55.56, 72.58) | 58.87  (51.55, 66.19) |  | 64.08  (57.40, 70.75) |  |
|  |  | week 9 | 71.67  (64.49, 78.85) | 59.90  (51.15, 68.65) | 0.062 | 70.58  (64.31, 76.84) | 0.786 |
|  | Emotion | Baseline | 58.08  (49.60, 66.56) | 66.93  (59.71, 74.15) |  | 69.02  (63.00, 75.04) |  |
|  |  | week 9 | 72.71  (65.35, 80.06) | 68.23  (61.34, 75.12) | **0.030*** | 71.31  (65.59, 77.03) | 0.234 |
|  | Cognitive | Baseline | 63.03  (56.79, 69.27) | 55.72  (48.79, 62.65) |  | 65.62  (59.93, 71.32) |  |
|  |  | week 9 | 75.66  (68.57, 82.76) | 59.90  (52.60, 67.20) | **0.010*** | 73.97  (67.34, 80.60) | 0.543 |
|  | Social | Baseline | 65.11  (55.90, 74.31) | 66.15  (57.59, 74.71) |  | 67.71  (57.59, 77.83) |  |
|  |  | week 9 | 75.53  (68.36, 82.69) | 69.28  (61.63, 76.93) | 0.180 | 75.95  (68.01, 83.88) | 0.978 |
| **Symptom scales** | | | | | | | |
|  | Fatigue | Baseline | 57.99  (51.17, 64.80) | 56.60  (49.44, 63.76) |  | 56.95  (50.13, 63.76) |  |
|  |  | week 9 | 39.84  (34.88, 44.80) | 54.52  (47.36, 61.67) | **<0.001*** | 41.23  (35.57, 46.89) | 0.695 |
|  | Nausea /  Vomiting | Baseline | 11.98  (5.83, 18.13) | 16.15  (9.59, 22.82) |  | 10.42  (5.19, 15.65) |  |
|  |  | week 9 | 9.95  (3.32, 16.58) | 13.55  (8.17, 18.93) | 0.753 | 6.29  (3.20, 9.37) | 0.366 |
|  | Pain | Baseline | 29.17  (20.81, 37.53) | 34.37  (26.16, 42.58) |  | 29.17  (21.69, 36.64) |  |
|  |  | week 9 | 19.87  (14.31, 25.42) | 28.12  (18.78, 37.46) | 0.222 | 22.15  (14.95, 29.35) | 0.592 |
|  | Dyspnea | Baseline | 30.20  (21.91, 38.48) | 22.90  (15.78, 30.02) |  | 24.98  (17.51, 32.46) |  |
|  |  | week 9 | 16.67  (10.65, 22.68) | 23.95  (15.74, 32.16) | **0.026*** | 20.63  (12.25, 29.01) | 0.258 |
|  | Sleeping disturbances | Baseline | 40.61  (32.11, 49.10) | 37.49  (27.95, 47.02) |  | 42.69  (32.46, 52.93) |  |
|  |  | week 9 | 29.75  (21.43, 38.07) | 37.49  (27.48, 47.50) | 0.126 | 33.35  (24.46, 42.24) | 0.629 |
|  | Appetite Loss | Baseline | 27.07  (17.69, 36.45) | 22.90  (15.16, 30.35) |  | 22.91  (14.58, 31.23) |  |
|  |  | week 9 | 12.41  (5.26, 19.55) | 23.94  (16.32, 31.57) | **0.005*** | 20.78  (11.93, 29.63) | 0.078 |
|  | Constipation | Baseline | 27.08  (15.89, 38.28) | 24.99  (15.83, 34.15) |  | 33.33  (24.69, 41.96) |  |
|  |  | week 9 | 22.62  (12.94, 32.30) | 22.90  (15.78, 30.17) | 0.685 | 21.39  (13.47, 29.31) | 0.221 |
|  | Diarrhea | Baseline | 18.74  (10.70, 26.78) | 29.16  (20.66, 37.66) |  | 18.74  (10.70, 26.78) |  |
|  |  | week 9 | 6.08  (0.48, 11.68) | 21.86  (12.91, 30.82) | **0.020*** | 14.71  (8.07, 21.34) | **0.019*** |
|  | Financial difficulties | Baseline | 17.69  (11.60, 23.78) | 28.11  (17.94, 38.29) |  | 26.03  (15.57, 36.49) |  |
|  |  | week 9 | 13.78  (7.11, 20.45) | 23.95  (13.27, 34.63) | 0.606 | 15.43  (7.50, 23.36) | 0.666 |

Data shown in mean (95% confidence interval). * p<0.05.

**Supplementary table 3. Safety-related outcomes**

|  | Mx group | | |  | UC group | | |  | SMx group | | |
| --- | --- | --- | --- | --- | --- | --- | --- | --- | --- | --- | --- |
|  | Baseline | Week 9 | *p* |  | Baseline | Week 9 | *p* |  | Baseline | Week 9 | *p* |
| AST  (U/L) | 24.47  (21.70, 27.24) | 26.23  (20.12, 32.33) | 0.374 |  | 24.72  (21.99, 27.44) | 24.66  (21.53, 27.79) | 0.946 |  | 23.41  (21.04, 25.77) | 23.10  (21.04, 25.16) | 0.609 |
| ALT  (U/L) | 18.59  (15.81, 21.37) | 18.74  (14.73, 22.75) | 0.925 |  | 21.28  (17.91, 24.65) | 20.81  (17.75, 23.88) | 0.539 |  | 18.56  (14.95, 22.17) | 18.53  (15.83, 21.23) | 0.708 |
| BUN  (mg/dL) | 13.32  (12.09, 14.54) | 13.41  (12.33, 14.50) | 0.772 |  | 14.43  (13.02, 15.84) | 15.56  (13.83, 17.29) | 0.169 |  | 14.03  (12.77, 15.29) | 14.10  (12.62, 15.58) | 0.779 |
| Creatinine  (mg/dL) | 0.756  (0.694, 0.817) | 0.740  (0.664, 0.815) | 0.496 |  | 0.777  (0.682, 0.872) | 0.786  (0.681, 0.891) | 0.663 |  | 0.761  (0.698, 0.824) | 0.740  (0.678, 0.802) | 0.130 |
| SBP  (mmHg) | 118.94  (113.56, 124.31) | 116.39  (111.40, 121.37) | 0.266 |  | 120.16  (114.34,125.98) | 119.63  (114.67,124.58) | 0.839 |  | 119.97  (114.93, 125.00) | 118.50  (113.51, 123.49) | 0.323 |
| DBP  (mmHg) | 74.34  (69.16, 79.52) | 72.26  (67.64, 76.88) | 0.396 |  | 71.66  (67.65, 75.66) | 72.25  (68.00, 76.50) | 0.795 |  | 72.97  (69.36, 76.57) | 73.67  (69.73, 77.61) | 0.913 |
| PR  (/min) | 76.59  (72.18, 81.01) | 80.06  (76.22, 83.91) | 0.154 |  | 72.16  (68.07, 76.25) | 72.06  (67.98, 76.15) | 0.951 |  | 74.28  (71.49, 77.07) | 76.00  (73.33, 78.68) | 0.390 |
| BT  (°C) | 36.51  (36.44, 36.58) | 36.48  (36.41, 36.54) | 0.434 |  | 36.50  (36.41, 36.59) | 36.59  (36.52, 36.67) | 0.066 |  | 36.52  (36.48, 36.58) | 36.53  (36.45, 36.61) | 0.813 |

Data shown as mean (95% Confidence Interval). AST: aspartate aminotransferase; ALT: alanine aminotransferase; BUN: blood urea nitrogen; SBP: systolic blood pressure; DBP: diastolic blood pressure; PR: pulse rate; BT: body temperature

**Supplementary table 4. Blood analysis results done before and after the 8-week interventions.**

|  | Moxibustion group | | | |  | Usual care group | | |  | Sham moxibustion group | | |
| --- | --- | --- | --- | --- | --- | --- | --- | --- | --- | --- | --- | --- |
|  | | Baseline | Week 9 | *p* |  | Baseline | Week 9 | *p* |  | Baseline | Week 9 | *p* |
| Total Bilirubin | 0.596 (0.490, 0.702) | | 0.514 (0.445, 0.583) | 0.027 |  | 0.554 (0.455, 0.653) | 0.567 (0.472, 0.662) | 0.687 |  | 0.539 (0.433, 0.645) | 0.533 (0.446, 0.620) | 0.780 |
| GGT | 20.94 (16.19, 25.69) | | 22.55 (15.41, 29.69) | 0.415 |  | 22.13 (15.79, 28.46) | 22.00 (14.25, 29.75) | 0.901 |  | 19.50 (15.22, 23.78) | 19.90 (15.62, 24.18) | 0.233 |
| Albumin | 4.40 (4.31, 4.49) | | 4.38 (4.28, 4.48) | 0.783 |  | 4.35 (4.25, 4.45) | 4.33 (4.23, 4.43) | 0.594 |  | 4.34 (4.24, 4.43) | 4.35 (4.22, 4.48) | 0.873 |
| Glucose | 105.31 (96.25, 114.37) | | 112.94 (98.45, 127.42) | 0.252 |  | 119.59 (104.03, 135.16) | 103.59 (96.17, 111.02) | 0.010 |  | 125.19 (107.41, 142.96) | 119.38 (104.51, 134.25) | 0.426 |
| HbA1c | 5.60 (5.45, 5.75) | | 5.57 (5.38, 5.76) | 0.663 |  | 5.61 (5.45, 5.77) | 5.53 (5.39, 5.67) | 0.066 |  | 5.95 (5.67, 6.23) | 5.88 (5.64, 6.13) | 0.486 |
| RBC | 429.78  (415.97, 443.59) | | 428.19  (413.82, 442.56) | 0.361 |  | 437.59  (421.35, 453.83) | 433.31  (417.47, 449.16) | 0.232 |  | 425.75  (398.31, 453.19) | 435.48  (415.32, 455.65) | 0.389 |
| WBC | 52.94 (47.20, 58.68) | | 53.98 (47.64, 60.33) | 0.648 |  | 52.01 (46.83, 57.20) | 52.43, 48.13, 56.73) | 0.842 |  | 53.73 (50.81, 56.66) | 54.64 (50.40, 58.87) | 0.573 |
| Neutrophil | 54.06 (50.28, 57.83) | | 53.40 (50.45, 56.35) | 0.608 |  | 56.28 (52.96, 59.61) | 56.38 (52.80, 59.95) | 0.942 |  | 57.48 (53.85, 61.11) | 56.59 (53.74, 59.45) | 0.726 |
| Monocyte | 8.62 (5.17, 12.06) | | 7.05 (6.52, 7.59) | 0.323 |  | 7.17 (6.31, 8.03) | 7.37 (6.57, 8.17) | 0.408 |  | 7.08 (6.32, 7.83) | 7.24 (6.59, 7.89) | 0.290 |
| Lymphocyte | 34.50 (30.81, 38.20) | | 36.95 (33.84, 40.05) | 0.067 |  | 33.25 (30.03, 36.47) | 33.08 (29.97, 36.18) | 0.882 |  | 32.67 (29.46, 35.88) | 33.30 (30.44, 36.15) | 0.905 |
| ANC | 2902 (2487, 3316) | | 2912 (2495, 3330) | 0.938 |  | 3021 (2687, 3355) | 2992 (2628, 3356) | 0.864 |  | 3083 (2837, 3329) | 3084 (2780, 3389) | 0.842 |
| Hemoglobin | 13.04 (12.68, 13.40) | | 13.00 (12.62, 13.38) | 0.437 |  | 13.34 (12.86, 13.83) | 13.18 (12.71, 13.65) | 0.088 |  | 12.90 (12.41, 13.39) | 13.01 (12.45, 13.56) | 0.290 |
| Hematocrit | 39.93 (38.80, 41.07) | | 39.85 (38.68, 41.02) | 0.557 |  | 40.42 (39.08, 41.75) | 40.18 (38.85, 41.52) | 0.503 |  | 39.64 (38.15, 41.12) | 39.62 (37.99, 41.26) | 0.966 |
| Platelet | 221.03  (205.65, 236.41) | | 223.13  (205.02, 241.23) | 0.457 |  | 216.56  (197.36, 235.76) | 216.78  (194.35, 239.22) | 0.963 |  | 232.31  (211.63, 252.99) | 239.34  (216.30, 262.39) | 0.536 |
| ESR | 15.90 (12.33, 19.48) | | 14.42 (11.06, 17.78) | 0.199 |  | 11.41 (8.72, 14.09) | 11.94 (8.82, 15.06) | 0.546 |  | 14.09 (9.58, 18.61) | 13.66 (9.15, 18.16) | 0.774 |
| CRP | 0.258 (0.060, 0.457) | | 0.090 (0.052, 0.128) | 0.099 |  | 0.159 (0.062, 0.256) | 0.114 (0.054, 0.173) | 0.409 |  | 0.251 (0.113, 0.388) | 0.186 (-0.026, 0.399) | 0.442 |
| TSH | 2.18 (1.57, 2.79) | | 2.28 (1.70, 2.85) | 0.808 |  | 1.89 (1.65, 2.12) | 2.00 (1.68, 2.33) | 0.421 |  | 1.71 (1.28, 2.14) | 1.78 (1.40, 2.17) | 0.837 |
| T4 | 1.21 (1.13, 1.30) | | 1.20 (1.12, 1.29) | 0.653 |  | 1.14 (1.09, 1.19) | 1.16 (1.10, 1.22) | 0.464 |  | 1.21 (1.12, 1.31) | 1.19 (1.10, 1.28) | 0.474 |

Data shown in mean (95% confidence interval). GGT: gamma-glutamyl transferase; HbA1c: hemoglobin A1c; RBC: red blood cell count; WBC: white blood cell count; ANC: absolute neutrophil count; ESR: erythrocyte sedimentation rate; CRP: C-reactive protein; TSH: thyroid-stimulating hormone
